# Plasma Beta-Hydroxybutyrate and All-Cause Mortality in Patients with Liver Cirrhosis

**DOI:** 10.3390/biomedicines13051120

**Published:** 2025-05-06

**Authors:** Mateo Chvatal-Medina, Yakun Li, María Camila Trillos-Almanza, Adrian Post, Margery A. Connelly, Han Moshage, Stephan J. L. Bakker, Vincent E. de Meijer, Hans Blokzijl, Robin P. F. Dullaart

**Affiliations:** 1 Department of Gastroenterology and Hepatology, University Medical Center Groningen, University of Groningen, P.O. Box 30.001, 9700 RB Groningen, The Netherlands; 2 Department of Internal Medicine, Division of Nephrology, University Medical Center Groningen, University of Groningen, P.O. Box 30.001, 9700 RB Groningen, The Netherlands; 3 Labcorp, 100 Perimeter Park, Morrisville, NC 27560, USA; 4 Department of Surgery, Division of Hepato-Pancreato-Biliary Surgery and Liver Transplantation, University Medical Center Groningen, University of Groningen, P.O. Box 30.001, 9700 RB Groningen, The Netherlands; 5 Department of Internal Medicine, Division of Endocrinology, University Medical Center Groningen, University of Groningen, P.O. Box 30.001, 9700 RB Groningen, The Netherlands

**Keywords:** cirrhosis, β-hydroxybutyrate, ketone bodies, NMR spectroscopy, Prevention of Renal and Vascular End-stage Disease (PREVEND) cohort study, TransplantLines cohort and biobank study

## Abstract

**Background:** Liver cirrhosis is often accompanied by metabolic dysfunction. Circulating β-hydroxybutyrate (BHB), the most abundant ketone body, is an emerging metabolic biomarker of mitochondrial dysfunction. **Methods:** In this prospective observational study, we evaluated plasma BHB concentrations in patients with cirrhosis compared to the general population and investigated their association with all-cause mortality in cirrhosis. Plasma BHB, measured by nuclear magnetic resonance spectroscopy, was compared between 125 patients with cirrhosis on the waiting list for liver transplantation (TransplantLines cohort study; NCT03272841) with 125 propensity-score-matched participants from the population-dwelling PREVEND cohort. Associations of BHB with all-cause mortality were established by tertile-based log-rank tests and Cox regression analyses. A generalized additive model was fitted to assess a potential non-linear association between BHB and mortality. **Results:** Patients with cirrhosis had lower plasma BHB concentrations than matched PREVEND participants (111.5 µmol/L vs. 138.4 µmol/L, *p* = 0.02). During 133 (interquartile range 42–375) days of follow up, 27 patients died. All-cause mortality was lowest in the middle BHB tertile and highest in the upper BHB tertile (*p* < 0.001 by log-rank test). A non-linear, J-shaped association between BHB levels and mortality risk was found with a higher risk of death with the highest and lowest BHB levels. In Cox regression analyses, adjusted for age, sex, MELD score, diabetes, and HDL cholesterol, mortality was highest in the highest BHB tertile (T3 vs. T2 HR: 7.6, 95% CI: 2.3–25.6, *p* < 0.001). Mortality also tended to be higher in the lowest vs. the middle (T1 vs. T2 HR: 3.5, 95% CI: 0.9–11.7, *p* = 0.06). Sensitivity analyses, excluding diabetic patients and those with metabolic dysfunction-associated steatotic liver disease, confirmed the robustness of these findings. **Conclusion:** BHB levels exhibit a J-shaped association with the risk of death in patients with liver cirrhosis. The highest circulating BHB levels are independently associated with increased mortality risk, potentially reflecting underlying metabolic dysregulation. Future studies are necessary to validate the utility of BHB as a prognostic target in cirrhosis.

## 1. Introduction

Liver cirrhosis represents a significant global health burden that is responsible for 4% of all deaths and encompasses a spectrum of etiologies that ultimately lead to liver failure [1,2]. Liver transplantation (LT) is the only curative therapy for patients with acute and chronic end-stage liver disease. Much effort has been made to characterize which patients benefit the most from LT, with widespread scoring systems such as the Child–Turcotte-Pugh (CTP) classification and the Model for End-Stage Liver Disease (MELD) score [3,4,5]. However, these scoring systems primarily assess liver function and transplant eligibility, rather than taking account of the substantial cardiovascular and metabolic burden in cirrhosis [6,7]. The rising prevalence of cardiometabolic comorbidities, including type 2 diabetes, cardiovascular disease, hypertension, and chronic kidney disease, further complicates risk assessment and management in cirrhosis [8,9,10,11,12,13]. Given these limitations, there is a need to identify novel biomarkers that better reflect systemic metabolic dysfunction and improve risk stratification in liver disease.

Mitochondrial dysfunction is a metabolic hallmark of advanced liver disease [14,15]. Among its different molecular pathways, mitochondrial metabolism is flexible and uses different substrates for energy generation. One such process is ketogenesis, which takes place in perivenous hepatocytes and converts fatty acids into ketone bodies, namely β-hydroxybutyrate (BHB), acetoacetate and acetone [16,17]. After triglycerides are broken down, free fatty acids are taken up by hepatocytes and metabolized in the mitochondria through β-oxidation, thereby producing ATP and acetyl-CoA. Under certain metabolic conditions, acetyl-CoA cannot enter the citric acid cycle and instead accumulates. It is then converted into acetoacetate via acetoacetyl-CoA and hydroxy-methylglutaryl-CoA [17]. Most acetoacetate is reduced to BHB, the main plasma ketone body, while a small part is broken down to acetone [16,17]. Since ketone production occurs in hepatocyte mitochondria, these pathways may serve as useful markers of metabolic dysfunction in cirrhosis.

There is no conclusive evidence yet on the effects of impaired liver function on circulating ketone bodies, especially BHB [18]. Most available evidence comes from the specific context of metabolic dysfunction-associated steatotic liver disease (MASLD), but reports have been inconsistent, showing increased, normal, and decreased levels of ketone bodies in patients with MASLD [19,20,21,22]. In the present prospective observational study, we aimed to (i) compare BHB plasma levels in patients with cirrhosis with those of general population, (ii) examine its potential association with clinical and laboratory variables, and (iii) investigate its association with all-cause mortality.

## 2. Materials and Methods

### 2.1. Study Design

This study was conducted following the STROBE (Strengthening the Reporting of Observational Studies in Epidemiology) guidelines. Patients with cirrhosis were selected from the TransplantLines cohort, a comprehensive prospective observational study conducted at the University Medical Center Groningen (UMCG), The Netherlands (NCT03272841) [23]. The TransplantLines study follows solid organ transplant recipients and donors and was approved by the UMCG Medical Ethics Committee (METc 2014/077). All study procedures adhered to the principles of the Declaration of Helsinki [24]. Patients who were waitlisted for LT by the hospital transplant committee until June 2021 were included in the study. Exclusion criteria included the inability to understand the Dutch language, cognitive impairments preventing the completion of questionnaires and participation in physical assessments, as well as a lack of ketone body measurements (Supplementary Figure S1).

For the reference group, data were obtained from the PREVEND (Prevention of Renal and Vascular End-stage Disease) study, a large population-based cohort in the city of Groningen, The Netherlands, which has been previously described elsewhere [25]. The PREVEND study was approved by the UMCG Medical Ethics Committee (MEC96/01/022) and conducted according to the Declaration of Helsinki. Briefly, all residents of the city of Groningen, The Netherlands, aged 28 to 75 years were invited to submit a morning urine sample and complete a demographic and cardiovascular health questionnaire. Pregnant individuals and those with insulin-dependent diabetes were excluded to prevent confounding by gestational changes and changes in metabolism due to type 1 diabetes. From the first screening, all individuals with a urinary albumin concentration ≥ 10 mg/L, along with a randomly selected control group with urinary albumin < 10 mg/L, were invited for further evaluation in an outpatient clinic. This study includes PREVEND participants from the second screening round (2001–2003) who had available ketone body measurements and no history of liver disease. All participants provided written informed consent to participate in the TransplantLines and PREVEND studies.

### 2.2. Data Collection and Clinical Measurements

Data from the TransplantLines cohort were collected from June 2015 until June 2021 for these analyses. Outpatient visits involved standardized clinical assessments, questionnaire administration, and blood and urine sample collection in accordance with the TransplantLines protocol [23]. Clinical and demographic data were obtained through patient interviews and verified against electronic hospital records. Extracted medical information included comorbidities, history of cardiovascular disease (CVD), diabetes (determined by fasting glucose concentrations of ≥7.0 mmol/L, non-fasting plasma glucose of >11.1 mmol/L, HbA1c > 6.5% or self-reported diagnosis), medication use (glucose- and lipid-lowering drugs, antihypertensive medication), hospital admissions, mortality status, and cirrhosis etiology. The MELD score was calculated based on serum total bilirubin, creatinine, and the international normalized ratio (INR) [5]. The CTP classification was determined using total bilirubin, serum albumin, INR, ascites presence, and hepatic encephalopathy status [3]. Anthropometric measurements, including weight, height, body mass index (BMI), and blood pressure (BP), were recorded as per the TransplantLines protocol. Hypertension is defined as systolic BP > 140 mmHg, diastolic BP > 90 mmHg, or antihypertensive drug use. Alcohol consumption was recorded as grams per day, with one drink assumed to contain 10 g of alcohol. For the PREVEND cohort, demographic data, medical history (CVD, diabetes, renal disease), and medication use were collected through standardized questionnaires and were supplemented with a pharmacy-dispensing registry, as per the original cohort design [25,26]. Participants maintained their regular medication during sample collection. Venous blood samples were collected in both the TransplantLines and PREVEND cohorts after an overnight fast. Data on mortality were extracted from electronic records and confirmed with reports from the Dutch Central Bureau of Statistics.

### 2.3. Laboratory Analysis

Aliquots of EDTA-plasma were obtained by centrifugation at 1400× *g* for 15 min at 4 °C and frozen at −80 °C until analysis.

In the TransplantLines cohort, standardized biochemical methods were used to analyze serum alanine aminotransferase (ALT), aspartate aminotransferase (AST), gamma-glutamyl transferase (GGT), alkaline phosphatase (ALP), total bilirubin, albumin, serum creatinine, hemoglobin, thrombocytes, leukocytes, glycated hemoglobin (HbA1c), and plasma glucose as described [27,28]. eGFR was measured using the creatinine-based CKD-EPI equation [29]. For the PREVEND cohort, laboratory procedures have been extensively described [26,30]. All these measurements were performed at the Department of Laboratory Medicine, University Medical Center Groningen (UMCG), The Netherlands.

To measure BHB, plasma samples from the TransplantLines and PREVEND cohorts were shipped to Labcorp (Morrisville, NC, USA). Samples were analyzed using high-throughput nuclear magnetic resonance (NMR) spectroscopy, as previously described in detail [20,31]. In brief, plasma BHB was measured using the Vantera^®^ Clinical Analyzer (LabCorp), a fully automated, high-throughput, 400 MHz proton (^1^H) NMR platform. Samples were prepared on board the instrument and delivered to the flow probe in the magnetic field automatically. BHB signals were represented in the spectral region between 1.13 and 1.19 ppm, which was then deconvoluted into its parts, and background signals were subtracted. The concentration was ultimately determined based on the peak area from a standard with known concentration [31]. For BHB, the coefficients of variation for intra-assay and inter-assay precision were 1.3% and 9.3%, respectively. Total cholesterol, high-density lipoprotein (HDL) cholesterol, and triglycerides were also measured by NMR spectroscopy with the Vantera^®^ Clinical Analyzer using the previously reported LP4 algorithm [32].

### 2.4. Statistical Analysis

All statistical analyses were performed using R software (version 4.2.1, R Foundation for Statistical Computing, Vienna, Austria). A two-sided *p*-value < 0.05 was considered statistically significant. Continuous variables were expressed as medians with interquartile ranges (IQRs). Categorical variables were reported as counts and percentages. For continuous variables, Mann–Whitney U-tests were applied. For comparisons across tertiles, the Kruskal–Wallis test was used. Chi-square tests were used for categorical variables. To minimize confounding by comparing patients with cirrhosis with PREVEND participants, propensity score matching (PSM) at a 1:1 ratio was performed using an optimal matching algorithm based on age, sex, BMI, history of diabetes, and history of cardiovascular disease.

Univariable linear regression analyses were carried out to assess the association between clinical and laboratory variables and BHB. Beta-coefficients, 95% confidence intervals (CIs), and *p*-values were reported. Multivariable regression analyses were subsequently conducted and reported similarly. Model assumptions, including linearity, homoscedasticity, and independence of residuals, were assessed through residual diagnostics. To minimize multicollinearity, only variables with a variance inflation factor (VIF) below five were retained in the multivariable regression model.

Kaplan–Meier survival curves with log-rank tests were generated to compare survival across BHB tertiles. Deviations from linearity were tested by comparing linear models with nonlinear models using natural splines with three degrees of freedom, and the likelihood ratio test (LRT) determined the model with a better fit. A generalized additive model (GAM) and a locally estimated scatterplot smoothing (LOESS) curve were used to visualize non-linear relationships between variables. Crude and multivariable-adjusted Cox proportional hazard regression models were computed to investigate the relationship between BHB levels and overall mortality, using tertile-based BHB categories as the exposure variable. The proportional hazards assumption was evaluated using Schoenfeld residuals. Adjustments were made for a priori selected variables, including age, sex, history of diabetes, MELD score and HDL-cholesterol. Sensitivity analyses were performed excluding patients with diabetes and separately excluding those with MASLD as the etiology of cirrhosis. The discriminatory performance for 90-day mortality was assessed by calculating the area under the receiver operating characteristic curve (AUC-ROC) with 95% confidence intervals using DeLong’s method.

## 3. Results

### 3.1. Baseline Clinical and Laboratory Characteristics Between Patients with Cirrhosis and PREVEND Participants

Data from 125 subjects with liver cirrhosis from the TransplantLines cohort and 4833 participants from the community-dwelling Prevention of Renal and Vascular End-stage Disease (PREVEND) cohort were available for this study (Table S1). Patients with cirrhosis showed a trend of lower BHB (111.5 µmol/L vs. 121.8 µmol/L, *p* = 0.1). Participants with cirrhosis differed from PREVEND participants with respect to most demographic and clinical characteristics at baseline (Table S1).

Given the substantial baseline differences between patients with cirrhosis and PREVEND participants (Table S1), a propensity score matching (PSM) procedure was carried out, adjusting for age, sex, BMI, history of diabetes, and history of cardiovascular disease. This PSM analysis included 125 patients with cirrhosis and 125 PREVEND participants (Table 1). In the matched subset, age, sex distribution, history of diabetes, and cardiovascular disease were not different between the groups. The use of antihypertensive and glucose-lowering medication was still more frequent in the patients with cirrhosis. BHB was lower in the patients with cirrhosis (111.5 µmol/L vs. 138.4 µmol/L, *p* = 0.02), as were plasma lipids and lipoproteins (Table 1). Transaminases, alkaline phosphatase, and total bilirubin were higher, whereas hemoglobin was lower in patients with cirrhosis.

**Figure 1:**
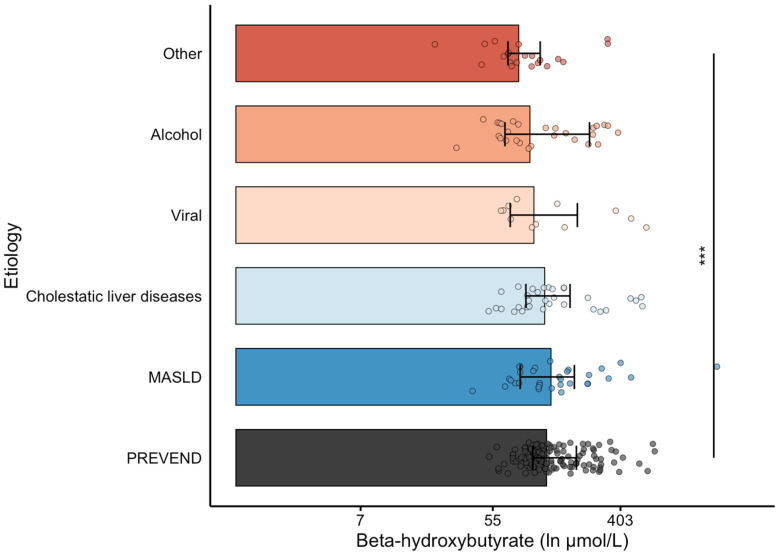
Ln-transformed β-hydroxybutyrate levels in cirrhosis by etiology of liver disease. The x-axis is in the ln-scale, with markers labeled in absolute values (µmol/L) for reference (i.e., e^2^ ≈ 7, e^4^ ≈ 55, e^6^ ≈ 403). A propensity-matched subset of PREVEND participants is included as a reference group. Statistical comparisons are indicated: *** *p* < 0.001 vs. the PREVEND subset. ”Other” etiologies refer to cases of autoimmune hepatitis, storage disease, biliary atresia, and vascular disease (portal vein thrombosis, sinusoidal obstruction, Budd–Chiari syndrome).

When comparing BHB according to cirrhosis etiology with PSM-selected PREVEND participants, BHB levels were lower among patients with cirrhosis with autoimmune hepatitis, storage diseases, and vascular diseases (including portal vein thrombosis, sinusoidal obstruction syndrome, and Budd–Chiari syndrome) as etiology (Figure 1).

### 3.2. Baseline Characteristics of Patients with Cirrhosis According to β-Hydroxybutyrate Levels

To examine the potential associations between BHB and clinical characteristics, patients with cirrhosis were divided into tertiles based on their plasma BHB concentrations (Table 2). No significant differences were seen across tertiles with respect to age, sex, etiologies of cirrhosis, renal function, BMI and blood pressure, history of CVD and diabetes, as well as in the use of antihypertensive, glucose-lowering medication, and lipid-lowering drugs. Alcohol use was slightly lower in the highest BHB tertile. Patients with the highest BHB levels had lower HDL cholesterol and higher triglycerides and total bilirubin levels, but no differences were seen between BHB tertiles in HbA1c and fasting glucose. There were also no significant differences in CTP classification and MELD scores between BHB tertiles.

Univariable regression analysis showed a positive association between BHB and history of diabetes and a negative association with HDL cholesterol. In multivariable analyses, both variables held a similar association (Table S2).

### 3.3. Longitudinal Analyses of β-Hydroxybutyrate with Overall Mortality in Patients with Cirrhosis

The 125 patients with cirrhosis were followed up with a median follow-up time of 133 days (IQR 42–375; maximum 1072). Overall, there were 27 deaths, corresponding to 21.6% (17 males and 10 females).

**Figure 2:**
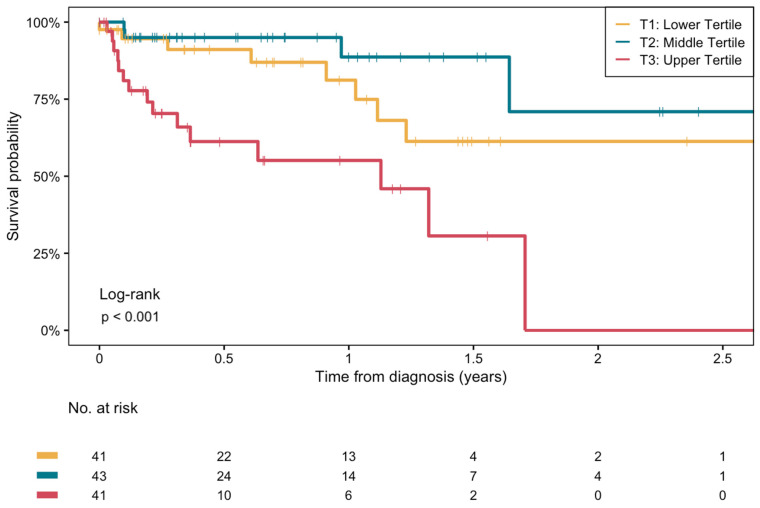
Kaplan–Meier survival curves for the association between plasma β-hydroxybutyrate concentrations and the risk of all-cause mortality in end-stage liver disease patients on the waiting list for LT. Log-rank test between middle and upper tertile: *p* < 0.001. Log-rank test between lower and upper tertile: *p* = 0.009. Log-rank test between lower and middle tertile: *p* = 0.2.

Figure 2 shows the Kaplan–Meier survival curves stratified by tertiles of plasma BHB concentrations. Mortality was highest in the highest tertile (T3) of BHB (overall log-rank test: *p* < 0.001). A statistically significant difference was noted between the middle tertile (T2) and T3 (log-rank test *p* < 0.001). The difference between the lowest tertile (T1) and T3 was also significant (log-rank test *p* = 0.009), while the difference in mortality between T1 and T2 did not reach significance (log-rank test *p* = 0.2). There were no significant differences in survival among different etiologies of cirrhosis (log-rank test *p* = 0.75, Supplementary Figure S2).

**Figure 3:**
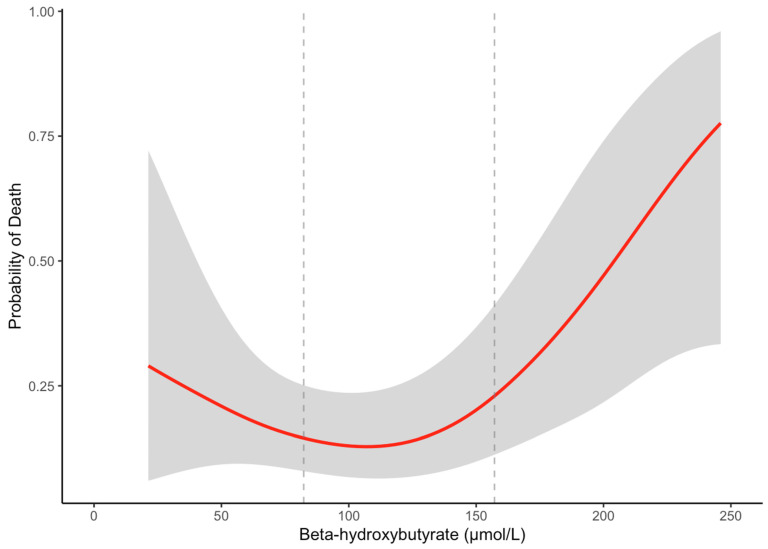
GAM-smoothed curve (with 95% confidence intervals) showing the relationship between β-hydroxybutyrate and the probability of overall death in patients with cirrhosis. The vertical dashed lines at 82.3 μmol/L and 157.2 μmol/L represent the tertile cutoffs for BHB.

We next tested for non-linearity in the association between BHB levels and survival using natural splines (df = 3). The likelihood ratio test showed a significant improvement in model fit with the non-linear model compared to the linear model (χ^2^ = 7.52, df = 2, *p* = 0.02), indicating a non-linear relationship between BHB and survival. We visualized this association using a generalized additive model (GAM) and locally estimated scatterplot smoothing (LOESS). Both crude models revealed a J-shaped association between BHB levels and overall mortality (Figure 3, Supplementary Figure S3). The risk of death was higher at both lower and higher BHB concentrations, with a nadir near the median value of BHB (~115 µmol/L).

Cox proportional hazard regression analyses were subsequently performed to assess the risk of overall death according to BHB levels, stratified in tertiles (Table 3). T2 was set as the reference tertile since the nadir of BHB with mortality was within the T2 range of values in the smoothed curves. A positive association was noted between BHB levels in T3 and increased mortality, compared to T2 (Model 1). The association remained significant after adjusting for age, sex, history of diabetes, the MELD score and HDL cholesterol (Model 5) (HR: 7.6, 95% CI: 2.3–25.6: *p* = 0.001) a in fully adjusted analysis. A sensitivity analysis excluding patients with a history of diabetes showed a sustained association between higher BHB levels in T3 and increased risk of mortality, compared to T2, both in crude and fully adjusted models (Models 1–4’). Further sensitivity analyses, excluding patients with MASLD and patients with rare etiologies of cirrhosis (i.e., vascular etiologies, autoimmune hepatitis, storage diseases and biliary atresia), showed a comparable association in all models (Table 3, Table S3). There was a trend of a higher risk of death in T1 compared to T2, in both crude and adjusted models (HR: 3.3, 95% CI: 0.9–11.7: *p* = 0.06 in fully adjusted analysis), which was also found in sensitivity analyses, excluding diabetes and MASLD. In exploratory analyses comparing T3 with T1, we also noticed a higher risk of death in T3 compared to T1, in both crude and partially adjusted models, and in sensitivity analyses (Table S4). This suggested that despite the non-linear association between BHB and mortality risk, the highest BHB levels were associated with the highest risk of death.

To explore the utility of BHB to discriminate short-term mortality, we generated 90-day, time-dependent ROC curves from the linear predictors of three models: (i) BHB tertiles alone, (ii) MELD score alone, and (iii) MELD score and BHB tertiles. The BHB model yielded an AUC of 0.70 (95% CI: 0.55–0.85, compared to the MELD model’s 0.77 (95% CI: 0.61–0.92) and the combined MELD + BHB model’s 0.79 (95% CI: 0.63–0.95) (Supplementary Figure S4).

## 4. Discussion

This study is, to the best of our knowledge, the first to comprehensively study and compare plasma BHB concentrations in patients with cirrhosis to a large community-dwelling cohort. We have demonstrated that BHB, the most abundant circulating ketone body, is lower in subjects with cirrhosis compared to the general population. Notably, a J-shaped pattern was found between BHB levels and mortality risk. In adjusted time-to event analysis, the highest levels of circulating BHB were associated with an increased risk of all-cause mortality, independent of the MELD score and diabetes history, and also in sensitivity analyses, excluding diabetic patients or MASLD as the etiology of cirrhosis. This association was also retained after excluding rare etiologies (vascular, autoimmune, biliary atresia, and storage diseases). Finally, BHB could hold promise in the clinical setting, with a fair discrimination for short-term mortality assessed through AUC-ROC.

The median plasma BHB concentrations in patients with cirrhosis amounted to 111.5 µmol/L, which was about 20% lower than in participants from the population-based PREVEND participants applying a propensity score matching procedure. Given the disparity in clinical characteristics, including a high diabetes prevalence, we considered it necessary to control for potential confounding because of the impact of metabolic dysfunction in diabetes on ketone metabolism, as well as the effects of glucose-lowering medications like metformin, among other variables [33,34]. In physiological conditions, ketogenesis processes free fatty acids in the mitochondria of hepatocytes at a proportional rate to total fat oxidation, under the influence of insulin as the main inhibitor and glucagon as the main stimulator [18,20]. Nonetheless, the progression of liver disease and hepatic tissue inflammation that results in abnormal liver function impacts the mitochondrial function directly and leads to a decline in energy production [35]. Possible explanations of this include the altered extrahepatic ketone metabolism, especially in the context of cirrhosis-related sarcopenia and cardiovascular dysfunction, as well as stress-induced ketogenesis driven by systemic inflammation [36,37]. Consequently, we hypothesize that the overall reduction in BHB concentrations in patients with cirrhosis from our sample, compared to PREVEND participants, is a result of this disrupted mitochondrial function and ketogenesis in cirrhotic livers.

Although BHB was not significantly related to the MELD score and the CTP classification, it was inversely associated with HDL cholesterol, which is known to decline progressively with more advanced cirrhosis [38]. Of various etiologies, we found BHB to be particularly lower in patients with underlying autoimmune hepatitis, storage disease, and vascular diseases (including portal vein thrombosis, sinusoidal obstruction syndrome, and Budd–Chiari) combined (“Other” etiologies). We hypothesize that this might be explained at least in part due to the profound parenchymal loss and disturbed hepatic architecture, altered mitochondrial function, and altered portal flow in these etiologies, which have an impact on ketogenesis [39,40,41,42,43]. Of note, BHB levels were unrelated to MASLD as the underlying etiology of cirrhosis. Yet, metabolic dysfunction in MASLD confers increased free fatty acid influx and lipogenesis, which drives ketogenesis and increases the concentration of circulating ketones like BHB [18,44]. Thus, higher BHB in MASLD per se would conceivably oppose lower BHB in more advanced fibrosis. As inflammatory damage progresses, there is a decrease in hepatic oxidative metabolism, which suggests that simple steatosis would drive the higher production of BHB, but as steatohepatitis worsens, BHB also decreases [45].

Overall positive effects of ketone bodies have been reported in cardiovascular health in the context of neuroprotection and their anti-inflammatory effects [46,47,48]. In the context of liver disease, some evidence suggests a protective role of BHB from alcohol-induced liver injury [49]. Still, evidence has linked higher ketone bodies with an increased risk of death in specific disorders including MASLD [20]. We found a J-shaped association between circulating BHB and all-cause mortality. The association of BHB with mortality remained significant after adjustment for the MELD score, diabetes status, and HDL cholesterol, as well as in sensitivity analyses, excluding diabetic patients or MASLD as the etiology of cirrhosis. These results indicate that this association is not entirely explained by common concomitant disorders or distinct etiologies. The reason for such a non-linear association remains to be determined, but we surmise that patients with the highest BHB would fare worse due to more severe metabolic dysfunction, likely related to oxidative stress and alterations in the redox balance [50,51]. Overall, functional and architectural damage on mitochondria that result in lower BHB production, as well as grave metabolic dysregulation that results in higher BHB production, ultimately increase the risk of death. In line with an association of BHB, as a potential proxy of mitochondrial dysfunction, with mortality, we have recently found that circulating citrate, an established marker of mitochondrial dysfunction, is elevated in cirrhosis and predicts mortality in such patients [27]. However, the association between high BHB and mortality seems paradoxical given that patients with cirrhosis have overall lower BHB compared to the general population. Our findings do not clarify whether circulating ketone bodies, mainly BHB, are directly and causally related to the increased risk of death, or rather if they reflect an underlying mechanism of metabolic dysfunction.

Findings from large observational studies in general population also demonstrate a higher risk of mortality and major adverse cardiovascular events in people with higher circulating BHB or total ketone bodies [52,53]. Therefore, we concur with previous literature that although impaired liver function in patients with cirrhosis lowers BHB production, a higher concentration of BHB (relative to usual levels) resulting from an altered metabolic state confers an increased risk of mortality [54]. Although patients with cirrhosis are already at an increased risk of death, those with the highest BHB concentrations fare comparatively worse. Finally, there was a trend of increased risk of mortality in those with the lowest BHB concentrations compared to the middle tertile, which might be explained in part due to severe functional and architectural damage, but sample size may be insufficient to ascertain this trend. Taken together, these data support a two-limb model: (i) very low BHB in cirrhosis may reflect severe mitochondrial failure because of functional and architectural damage; (ii) very high BHB—whether in cirrhosis or in the general population—marks a catabolic-stress phenotype characterized by unopposed lipolysis, insulin resistance, and systemic inflammation.

Although this study shows promise in the utility of BHB for short-term mortality discrimination, future work must be carried out to conclude whether adding BHB to MELD, MELD-Na or MELD-3.0, as well as frailty scores, improves the net re-classification. Additionally, further studies should serially quantify BHB during acute decompensation and recompensation and evaluate interventions that modulate ketone metabolism (e.g., SGLT2 inhibition and targeted nutritional therapy) in randomized settings. Also, since BHB is a modifiable metabolic signal dependent on energy homeostasis, novel regenerative strategies might also modulate BHB itself. For instance, recent pre-clinical studies using hydrogel-based delivery of mesenchymal stem cells improved liver regeneration in cirrhotic models and boosted hepatic β-hydroxybutyrate, but further research is warranted on this subject [55,56].

Several methodological aspects of this study should be noted. Most importantly, a key strength in our study is that it is the first to systematically evaluate the impact of plasma BHB as the main ketone body in patients with cirrhosis compared to a large community-dwelling cohort, including its relationship with all-cause mortality. It is also a robust assessment of the association between this ketone body with clinical, laboratory, and medication-related factors, made possible by the design of the TransplantLines cohort and PREVEND cohort. Furthermore, ketone bodies were determined using a precise NMR spectroscopy assay. Notably, there are several limitations to be addressed as well. First, the sample size and number of cases restrict the precision of our results. Although the entire sample corresponds to patients with cirrhosis and listed for liver transplantation, etiologies are heterogeneous, and their variability also impacts the precision of our results. We acknowledge that it would be ideal to match for other relevant variables such as types of glucose-lowering medication or alcohol consumption, but this would lead to overfitting in our propensity score model. Hence, this subject warrants further investigation. Second, since this is an observational study and epidemiological in nature, only association between BHB and all-cause mortality can be established, precluding causality, and preventing the precise determination of underlying mechanisms of association. Third, given the predominant North European origin of patients in the study, further validations with different populations are warranted.

## 5. Conclusions

We found lower circulating BHB in patients with cirrhosis, in particular when cirrhosis is due to autoimmune hepatitis, storage disease or vascular disease. We suggest that BHB represents a valuable biomarker of metabolic dysfunction to assess disease severity in cirrhosis. The J-shaped association between BHB and mortality underscores the need for mechanistic studies that inquire into the role of ketone body metabolism as a marker of metabolic dysfunction. Future studies are necessary to validate the utility of BHB as a prognostic target in cirrhosis. Our findings also underscore the necessity to clarify whether safety concerns would outbalance the potential utility of a ketogenic diet in cirrhosis.

## Figures and Tables

**Table 1:** Clinical and laboratory characteristics in cirrhotic and PREVEND participants (propensity score-matched).

	Cirrhosis (n = 125)	PREVEND (n = 125)	*p*-Value
β-hydroxybutyrate (µmol/L)	111.5 [75.9, 178.1]	138.4 [97.7, 197.5]	0.02
Age (years)	60 [52, 65]	58 [50, 66]	0.6
Sex (Female, %)	42 (33.6)	42 (33.6)	1.0
BMI (kg/m^2^)	27.8 [24.8, 30.9]	27.8 [25.5, 31.0]	0.7
Current smoking (N, %)	16 (12.8)	25 (20.0)	0.2
Alcohol consumption (g/day, %)			<0.001
0	120 (96.0)	43 (34.4)	
0.1–10	5 (4.0)	27 (21.6)	
10–30	0 (0.0)	28 (22.4)	
>30	0 (0.0)	27 (21.6)	
Systolic Blood Pressure (mmHg)	115 [107, 130]	132 [117, 144]	<0.001
Diastolic Blood Pressure (mmHg)	65 [59, 75]	76 [69, 83]	<0.001
History of cardiovascular disease (N, %)	6 (4.8)	5 (4.0)	1.0
History of diabetes (N, %)	35 (28.0)	33 (26.4)	0.9
Antihypertensive drugs (N, %)	79 (63.2)	22 (17.6)	<0.001
Glucose-lowering drugs (N, %)	34 (27.2)	20 (16.0)	0.05
Lipid-lowering drugs (N, %)	19 (15.2)	20 (16.0)	1.0
Etiology (N, %)		-	NA
MASLD	32 (25.6)		
Cholestatic liver disease	32 (25.6)		
Alcohol	28 (22.4)		
Viral	12 (9.6)		
Other	21 (16.8)		
CTP classification (N, %)		-	NA
A	27 (21.6)		
B	62 (49.6)		
C	36 (28.8)		
Fasting glucose (mmol/L)	6.35 [5.03, 8.00]	5.10 [4.60, 6.20]	0.002
eGFR (mL/min/1.73 m^2^)	99.5 [76.1, 109.7]	88.3 [75.6, 99.9]	0.01
Total cholesterol (mmol/L)	3.26 [2.61, 4.14]	5.55 [4.95, 6.14]	<0.001
HDL cholesterol (mmol/L)	0.88 [0.59, 1.19]	1.16 [0.92, 1.34]	<0.001
LDL cholesterol (mmol/L)	1.81 [1.29, 2.25]	3.71 [3.09, 4.17]	<0.001
Triglycerides (mmol/L)	0.67 [0.46, 1.07]	1.30 [0.98, 1.72]	<0.001
ALT (U/L)	39 [28, 59]	21 [15, 28]	<0.001
AST (U/L)	54 [44, 83]	24 [20, 30]	<0.001
GGT (U/L)	95 [49, 151]	28 [18, 45]	<0.001
AP (U/L)	141 [99, 210]	65 [54, 82]	<0.001
Total Bilirubin (mmol/L)	40 [23, 94]	8 [6, 10]	<0.001
Hemoglobin (mmol/L)	6.9 [5.9, 7.8]	8.5 [8.0, 9.0]	<0.001

Data are expressed in median (IQ range) for continuous variables and in numbers (N) and percentages for categorical variables. AP: alkaline phosphatase, ALT: alanine aminotransferase, AST: aspartate aminotransferase, BMI: body mass index, CTP: Child–Turcotte Pugh, eGFR: estimated glomerular filtration rate, GGT: gamma-glutaryl transferase, HDL: high-density lipoprotein, LDL: low-density lipoprotein, MASLD: metabolic-dysfunction-associated steatotic liver disease, PREVEND: Prevention of Renal and Vascular End-stage Disease. NA: not available ”Other” etiologies refer to cases of autoimmune hepatitis, storage disease, biliary atresia, and vascular disease (portal vein thrombosis, sinusoidal obstruction, Budd–Chiari syndrome).

**Table 2:** Baseline characteristics of patients with cirrhosis according to tertiles of β-hydroxybutyrate.

	T1 (n = 41): ≤82.3 μmol/L	T2 (n = 43): 82.3–157.2 μmol/L	T3 (n = 41): >157.2 μmol/L	*p*-Value
β -hydroxybutyrate (µmol/L)	66.7 [59.4, 74.6]	111.5 [97.7, 131.1]	277.2 [182.4, 373.7]	
Age (years)	58 [48, 62]	61 [55, 66]	60.00 [55, 65]	0.1
Sex (Female, %)	13 (31.7)	15 (34.9)	14 (34.1)	1.0
BMI (kg/m^2^)	27.8 [26.2, 30.4]	27.8 [25.0, 31.8]	28.1 [24.3, 30.40]	0.5
Smoking (%)	7 (17.1)	5 (11.6)	4 (9.8)	0.6
Alcohol consumption (g/day, %)				0.05
0	37 (90.2)	43 (100)	40 (97.6)	
0.1–10	4 (9.8)	0 (0.0)	1 (2.4)	
10–30	0 (0.0)	0 (0.0)	0 (0.0)	
>30	0 (0.0)	0 (0.0)	0 (0.0)	
Systolic Blood Pressure (mmHg)	115 [108, 128]	119 [108, 134]	111 [104, 122]	0.3
Diastolic Blood Pressure (mmHg)	65 [58, 75]	66 [61, 75]	63 [57, 71]	0.4
History of cardiovascular disease (N, %)	1 (2.4)	3 (7.0)	2 (4.9)	0.6
History of diabetes (N, %)	10 (24.4)	14 (32.6)	11 (26.8)	0.7
Antihypertensive drugs (N, %)	23 (56.1)	29 (67.4)	27 (65.9)	0.5
Glucose-lowering drugs (N, %)	7 (17.1)	17 (39.5)	10 (24.4)	0.06
Lipid-lowering drugs (N, %)	4 (9.8)	11 (25.6)	4 (9.8)	0.07
Etiology (N, %)				0.3
MASLD	6 (14.6)	13 (30.2)	13 (31.7)	
Cholestatic liver disease	7 (17.1)	14 (32.6)	11 (26.8)	
Alcohol	12 (29.3)	6 (14.0)	10 (24.4)	
Viral	5 (12.2)	3 (7.0)	4 (9.8)	
Other	11 (26.8)	7 (16.2)	3 (7.3)	
CTP classification (N, %)				0.9
A	9 (22.0)	10 (23.3)	8 (19.5)	
B	22 (53.7)	19 (44.2)	21 (51.2)	
C	10 (24.4)	14 (32.6)	12 (29.3)	
MELD score	14 [10, 18]	14 [10, 17]	17 [11, 21]	0.07
HbA1c (%)	5.0 [4.7, 5.4]	5.5 [4.8, 6.3]	4.6 [4.0, 5.3]	0.3
Fasting glucose (mmol/L)	6.2 [5.5, 6.7]	6.7 [5.4, 8.0]	7.0 [4.6, 8.8]	0.8
eGFR (mL/min/1.73 m^2^)	101.8 [76.4, 113.8]	100.3 [85.3, 112.2]	97.6 [69.5, 102.9]	0.05
Total cholesterol (mmol/L)	3.28 [2.79, 4.14]	3.21 [2.73, 4.01]	2.95 [2.33, 3.98]	0.6
HDL cholesterol (mmol/L)	1.11 [0.80, 1.27]	0.83 [0.63, 1.12]	0.67 [0.16, 1.06]	0.004
LDL cholesterol (mmol/L)	1.99 [1.42, 2.33]	1.81 [1.24, 2.37]	1.76 [1.19, 2.22]	0.6
Triglycerides (mmol/L)	0.56 [0.37, 0.71]	0.85 [0.54, 1.17]	0.79 [0.50, 1.08]	0.02
ALT (U/L)	35 [25, 56]	39 [30, 47]	44 [32, 78]	0.2
AST (U/L)	50 [41, 59]	53 [37, 70]	81 [44, 113]	0.06
GGT (U/L)	90 [47, 142]	101 [52, 214]	96 [60, 132]	0.7
AP (U/L)	136 [109, 177]	142 [101, 217]	153 [87, 234]	0.9
Total Bilirubin (mmol/L)	30 [17, 53]	50 [26, 93]	63 [24, 229]	0.02
Albumin (g/L)	31 [27, 36]	30 [27, 35]	32 [28, 37]	0.9
Hemoglobin (mmol/L)	7.2 [6.2, 8.1]	6.8 [6.1, 7.2]	6.4 [5.4, 8.1]	0.4

Data are expressed in median (IQR) for continuous variables and in numbers (N) and percentages for categorical variables. *p*-values by Kruskal–Wallis test for numeric variables and Chi-squared test for categorical variables. ALT: alanine aminotransferase, AP: alkaline phosphatase, AST: aspartate aminotransferase, BMI: body mass index, CTP: Child–Turcotte–Pugh, eGFR: estimated glomerular filtration rate, GGT: gamma-glutamyl transferase, HbA1c: glycated hemoglobin, HDL: high-density lipoprotein, LDL: low-density lipoprotein, MASLD: metabolic-dysfunction-associated steatotic liver disease, MELD: model for end-stage liver disease. “Other” etiologies refer to cases of autoimmune hepatitis, storage disease, biliary atresia, and vascular disease (portal vein thrombosis, sinusoidal obstruction, Budd–Chiari syndrome).

**Table 3:** Cox regression analyses for associations between plasma BHB levels and the risk of all-cause mortality in patients with cirrhosis.

	T1 HR [95% CI]	T2 (Reference)	T3 HR [95% CI]
All patients with cirrhosis (n = 125, deaths = 27)			
Model 1	2.3 [0.7–7.5] *p* = 0.2	Reference	**6.6 [2.2–20.2]** ***p* < 0.001**
Model 2	2.5 [0.7–8.2] *p* = 0.1	Reference	**6.6 [2.2–20.3]** ***p* < 0.001**
Model 3	2.9 [0.8–9.8] *p* = 0.1	Reference	**6.5 [2.1–20.1]** ***p* = 0.001**
Model 4	2.9 [0.8–10.0] *p* = 0.1	Reference	**8.3 [2.5–27.6]** ***p* < 0.001**
Model 5	3.3 [0.9–11.7] *p* = 0.06	Reference	**7.6 [2.3–25.6]** ***p* = 0.001**
Sensitivity analysis: Excluding patients with diabetes (n = 90, deaths = 21)			
Model 1	1.5 [0.4–5.4] *p* = 0.5	Reference	**7.3 [2.2–24.3]** ***p* = 0.001**
Model 2	1.5 [0.4–5.3] *p* = 0.6	Reference	**6.5 [1.9–21.7]** ***p* = 0.002**
Model 3	2.2 [0.6–8.3] *p* = 0.3	Reference	**6.5 [1.8–23.0]** ***p* = 0.004**
Model 4′	2.5 [0.6–9.4] *p* = 0.2	Reference	**5.4 [1.5–20.0]** ***p* = 0.01**
Sensitivity analysis: Excluding patients with MASLD (n = 93, deaths = 19)			
Model 1	1.3 [0.4–5.0] *p* = 0.7	Reference	**5.4 [1.6–18.1]** ***p* = 0.007**
Model 2	1.5 [0.4–5.7] *p* = 0.6	Reference	**5.6 [1.6–19.0]** ***p* = 0.006**
Model 3	2.2 [0.5–9.1] *p* = 0.3	Reference	**5.3 [1.5–18.3]** ***p* = 0.008**
Model 4	1.9 [0.5–8.2] *p* = 0.4	Reference	**7.3 [1.9–27.6]** ***p* = 0.004**
Model 5	2.1 [0.5–8.8] *p* = 0.3	Reference	**6.4 [1.7–24.8]** ***p* = 0.007**

Statistically significant hazard ratios in each mode are shown in bold. Model 1: crude model. Model 2: adjusted for age and sex. Model 3: adjusted for age, sex and MELD score. Model 4: adjusted for age, sex, MELD score and history of diabetes. Model 5: adjusted for age, sex, MELD score, history of diabetes and HDL cholesterol. Model 4′: in sensitivity analysis excluding diabetic patients model 4 was adjusted for age, sex, MELD score and HDL cholesterol. T1–T3: tertiles 1–3, HR: hazard ratio, CI: confidence interval, MASLD: metabolic-dysfunction-associated steatotic liver disease.

## Data Availability

Anonymized raw data will be made available by the authors upon reasonable request.

## Supplementary Materials

The following supporting information can be downloaded at https://www.mdpi.com/article/10.3390/biomedicines13051120/s1. Figure S1: Participant selection flow-chart; Figure S2: Kaplan–Meier survival curves for the association between different etiologies of cirrhosis and the risk of all-cause mortality in end-stage liver disease patients on THE waiting list for LT; Figure S3: LOESS curve showing the relationship of BHB and the probability of death in patients with cirrhosis; Figure S4: Receiver operating characteristic (ROC) curves for predicting 90-day all-cause mortality in patients with cirrhosis, comparing discrimination performance between plasma beta-hydroxybutyrate (BHB) and MELD score; Table S1: Clinical and laboratory characteristics in cirrhotic and all PREVEND participants; Table S2: Univariable and multivariable linear regression analysis showing association between BHB and relevant clinical and laboratory parameters in patients with cirrhosis; Table S3: Cox regression analyses for associations between plasma BHB levels and the risk of all-cause mortality in patients with cirrhosis, excluding rare etiologies; Table S4: Cox regression analyses for associations between plasma BHB levels and the risk of all-cause mortality in cirrhotic patients, between T1 and T3. Supplementary File S1: List of collaborative authors of TransplantLines Investigators.

## Abbreviations

The following abbreviations are used in this manuscript:

ALP	Alkaline Phosphatase
ALT	Alanine Aminotransferase
AST	Aspartate Aminotransferase
BHB	Beta (β)-Hydroxybutyrate
BMI	Body Mass Index
BP	Blood Pressure
CI	Confidence Interval
CTP	Child–Turcotte–Pugh Classification
CVD	Cardiovascular Disease
eGFR	Estimated Glomerular Filtration Rate
GAM	Generalized Additive Model
GGT	Gamma-Glutamyl Transferase
HBA1C	Glycated Hemoglobin
HDL	High-Density Lipoprotein
HR	Hazard Ratio
IQR	Interquartile Range
LDL	Low-Density Lipoprotein
LT	Liver Transplantation
MASLD	Metabolic Dysfunction-Associated Steatotic Liver Disease
MELD	Model for End-Stage Liver Disease
NMR	Nuclear Magnetic Resonance
PREVEND	Prevention of Renal and Vascular End-stage Disease
PSM	Propensity Score Matching
SD	Standard Deviation
UMCG	University Medical Center Groningen

## References

[1] Devarbhavi H., Asrani S.K., Arab J.P., Nartey Y.A., Pose E., Kamath P.S. (2023). Global burden of liver disease: 2023 update. J. Hepatol..

[2] Huang D.Q., Terrault N.A., Tacke F., Gluud L.L., Arrese M., Bugianesi E., Loomba R.L. (2023). Global epidemiology of cirrhosis—Aetiology, trends and predictions. Nat. Rev. Gastroenterol. Hepatol..

[3] Pugh R.N., Murray-Lyon I.M., Dawson J.L., Pietroni M.C., Williams R. (1973). Transection of the oesophagus for bleeding oesophageal varices. Br. J. Surg..

[4] Lucey M.R., Furuya K.N., Foley D.P. (2023). Liver Transplantation. N. Engl. J. Med..

[5] Kamath P.S., Wiesner R.H., Malinchoc M., Kremers W., Therneau T.M., Kosberg C.L., D’Amico G., Dickson E.R., Kim W.R. (2001). A model to predict survival in patients with end-stage liver disease. Hepatol. Baltim. Md..

[6] Goldberg D., Mantero A., Newcomb C., Delgado C., Forde K., Kaplan D., John B., Nuchovich N., Dominguez B., Emanuel E. (2021). Development and Validation of a Model to Predict Long-Term Survival After Liver Transplantation. Liver Transplant. Off. Publ. Am. Assoc. Study Liver Dis. Int. Liver Transplant. Soc..

[7] El-Khateeb E., Darwich A.S., Achour B., Athwal V., Rostami-Hodjegan A. (2021). Review article: Time to revisit Child-Pugh score as the basis for predicting drug clearance in hepatic impairment. Aliment. Pharmacol. Ther..

[8] Kumar R., Priyadarshi R.N., Anand U. (2021). Chronic renal dysfunction in cirrhosis: A new frontier in hepatology. World J. Gastroenterol..

[9] Fede G., Privitera G., Tomaselli T., Spadaro L., Purrello F. (2015). Cardiovascular dysfunction in patients with liver cirrhosis. Ann. Gastroenterol. Q. Publ. Hell. Soc. Gastroenterol..

[10] van den Berg E.H., Flores-Guerrero J.L., Gruppen E.G., de Borst M.H., Wolak-Dinsmore J., Connelly M.A., Bakker S.J.L., Dullaart R.P.F. (2019). Non-Alcoholic Fatty Liver Disease and Risk of Incident Type 2 Diabetes: Role of Circulating Branched-Chain Amino Acids. Nutrients.

[11] Cao L., An Y., Liu H., Jiang J., Liu W., Zhou Y., Shi M., Dai W., Lv Y., Zhao Y. (2024). Global epidemiology of type 2 diabetes in patients with NAFLD or MAFLD: A systematic review and meta-analysis. BMC Med..

[12] Chen S.H., Wan Q.S., Wang T., Zhang K.H. (2020). Fluid Biomarkers for Predicting the Prognosis of Liver Cirrhosis. Biomed. Res. Int..

[13] Thiele M., Villesen I.F., Niu L., Johansen S., Sulek K., Nishijima S., Van Espen L., Keller M., Israelsen M., Suvitaival T. (2024). Opportunities and barriers in omics-based biomarker discovery for steatotic liver diseases. J. Hepatol..

[14] Mansouri A., Gattolliat C.H., Asselah T. (2018). Mitochondrial Dysfunction and Signaling in Chronic Liver Diseases. Gastroenterology..

[15] Zhang I.W., Curto A., López-Vicario C., Casulleras M., Duran-Güell M., Flores-Costa R., Colsch B., Aguilar F., Aransay A.M., Lozano J.J. (2022). Mitochondrial dysfunction governs immunometabolism in leukocytes of patients with acute-on-chronic liver failure. J. Hepatol..

[16] Laffel L. (1999). Ketone bodies: A review of physiology, pathophysiology and application of monitoring to diabetes. Diabetes Metab. Res. Rev..

[17] Kolb H., Kempf K., Röhling M., Lenzen-Schulte M., Schloot N.C., Martin S. (2021). Ketone bodies: From enemy to friend and guardian angel. BMC Med..

[18] Bae J., Lee B.W. (2023). Association between Impaired Ketogenesis and Metabolic-Associated Fatty Liver Disease. Biomolecules.

[19] Lee S., Bae J., Jo D.R., Lee M., Lee Y.H., Kang E.S., Cha B.S., Lee B.W. (2023). Impaired ketogenesis is associated with metabolic-associated fatty liver disease in subjects with type 2 diabetes. Front. Endocrinol..

[20] Post A., Garcia E., van den Berg E.H., Flores-Guerrero J.L., Gruppen E.G., Groothof D., Daan Westenbrink B., Connelly M.A., Bakker S.J.l., Dullaart R.P.F. (2021). Nonalcoholic fatty liver disease, circulating ketone bodies and all-cause mortality in a general population-based cohort. Eur. J. Clin. Investig..

[21] Kim Y., Chang Y., Kwon M.J., Hong Y.S., Kim M.K., Sohn W., Cho Y.K., Shin H., Wild S.H., Byrne C.D. (2021). Fasting Ketonuria and the Risk of Incident Nonalcoholic Fatty Liver Disease With and Without Liver Fibrosis in Nondiabetic Adults. Am. J. Gastroenterol..

[22] Lim K., Kang M., Park J. (2021). Association between Fasting Ketonuria and Advanced Liver Fibrosis in Non-Alcoholic Fatty Liver Disease Patients without Prediabetes and Diabetes Mellitus. Nutrients.

[23] Eisenga M.F., Gomes-Neto A.W., van Londen M., Londen Mvan Ziengs A.L., Douwes R.M., Stam S.P., Osté M.C.J., Knobbe T.J., Hessels N.R., Buunk A.M. (2018). Rationale and design of TransplantLines: A prospective cohort study and biobank of solid organ transplant recipients. BMJ Open.

[24] World Medical Association (2013). World Medical Association Declaration of Helsinki: Ethical Principles for Medical Research Involving Human Subjects. JAMA.

[25] Diercks G.F.H., van Boven A.J., Hillege H.L., Janssen W.M.T., Kors J.A., de Jong P.E., Grobbee D.E., Crijns H.J., van Gilst W.H. (2000). Microalbuminuria is independently associated with ischaemic electrocardiographic abnormalities in a large non-diabetic population. The PREVEND (Prevention of REnal and Vascular ENdstage Disease) study. Eur. Heart J..

[26] Kappelle P.J.W.H., Gansevoort R.T., Hillege J.L., Wolffenbuttel B.H.R., Dullaart R.P.F., on behalf of the PREVEND study Group (2011). Apolipoprotein B/A-I and total cholesterol/high-density lipoprotein cholesterol ratios both predict cardiovascular events in the general population independently of nonlipid risk factors, albuminuria and C-reactive protein. J. Intern. Med..

[27] Li Y., Chvatal-Medina M., Trillos-Almanza M.C., Bourgonje A.R., Connelly M.A., Moshage H., Bakker S.J.L., de Meijer V.E., Blokzijl H., Dullaart R.P.F. (2024). Circulating Citrate Is Reversibly Elevated in Patients with End-Stage Liver Disease: Association with All-Cause Mortality. Int. J. Mol. Sci..

[28] Trillos-Almanza M.C., Chvatal-Medina M., Connelly M.A., Moshage H., Bakker S.J.L., de Meijder V.E., Blokzijl H., Dullaart R.P.F., TransplantLines Investigators (2024). Circulating Trimethylamine-N-Oxide Is Elevated in Liver Transplant Recipients. Int. J. Mol. Sci..

[29] Inker L.A., Schmid C.H., Tighiouart H., Eckfeldt J.H., Feldman H.I., Greene T., Kusek J.W., Manzi J., Van Lente F., Zhang Y.L. (2012). Estimating Glomerular Filtration Rate from Serum Creatinine and Cystatin C. N. Engl. J. Med..

[30] Gruppen E.G., Garcia E., Connelly M.A., Jeyarajah E.J., Otvos J.D., Bakker S.J.L., Dullaart R.P.F. (2017). TMAO is Associated with Mortality: Impact of Modestly Impaired Renal Function. Sci. Rep..

[31] Garcia E., Shalaurova I., Matyus S.P., Oskardmay D.N., Otvos J.D., Dullaart R.P.F., Connelly M.A. (2020). Ketone Bodies Are Mildly Elevated in Subjects with Type 2 Diabetes Mellitus and Are Inversely Associated with Insulin Resistance as Measured by the Lipoprotein Insulin Resistance Index. J. Clin. Med..

[32] Sokooti S., Flores-Guerrero J.L., Kieneker L.M., Heerspink H.J.L., Connelly M.A., Bakker S.J.L., Dullaart R.P.F. (2021). HDL Particle Subspecies and Their Association With Incident Type 2 Diabetes: The PREVEND Study. J. Clin. Endocrinol. Metab..

[33] van der Vaart A., Knol M.G.E., de Borst M.H., Bakker S.J.L., Connelly M.A., Garcia E., Bilo H.J.G., van Dijk P.R., Dullaart R.P.F. (2022). The Paradoxical Role of Circulating Ketone Bodies in Glycemic Control of Individuals with Type 2 Diabetes: High Risk, High Reward?. Biomolecules.

[34] Giaccari A., Solini A., Frontoni S., Del Prato S. (2021). Metformin Benefits: Another Example for Alternative Energy Substrate Mechanism?. Diabetes Care.

[35] Zhang I.W., López-Vicario C., Duran-Güell M., Clària J. (2021). Mitochondrial Dysfunction in Advanced Liver Disease: Emerging Concepts. Front. Mol. Biosci..

[36] Glass C., Hipskind P., Tsien C., Malin S.K., Kasumov T., Shah S.N., Kirwan J.P., Dasarathy S. (2013). Sarcopenia and a physiologically low respiratory quotient in patients with cirrhosis: A prospective controlled study. J. Appl. Physiol..

[37] Costa D., Simbrunner B., Jachs M., Hartl L., Bauer D., Paternostro R., Schwabl P., Scheiner B., Stättermayer A.F., Pinter M. (2021). Systemic inflammation increases across distinct stages of advanced chronic liver disease and correlates with decompensation and mortality. J. Hepatol..

[38] Berg EHvan den Flores-Guerrero J.L., Dullaart R.P.F. (2022). Lipoprotein Z, an abnormal LDL-like lipoprotein, independently predicts mortality in cirrhosis. Eur. J. Intern. Med..

[39] Li S., Niu M., Jing J., Huang Y., Zhang Z., Chen S., Shi G., He X., Zhang H., Xiao X. (2021). Metabolomic Signatures of Autoimmune Hepatitis in the Development of Cirrhosis. Front. Med..

[40] Gümüş E., Özen H. (2023). Glycogen storage diseases: An update. World J. Gastroenterol..

[41] Oo Y.H., Hubscher S.G., Adams D.H. (2010). Autoimmune hepatitis: New paradigms in the pathogenesis, diagnosis, and management. Hepatol. Int..

[42] Cazals-Hatem D., Vilgrain V., Genin P., Denninger M.H., Durand F., Belghiti J., Valla D., Degott C. (2003). Arterial and portal circulation and parenchymal changes in Budd–Chiari syndrome: A study in 17 explanted livers. Hepatol. Baltim. Md..

[43] Senzolo M., Garcia-Tsao G., García-Pagán J.C. (2021). Current knowledge and management of portal vein thrombosis in cirrhosis. J. Hepatol..

[44] Li Y., Yang P., Ye J., Xu Q., Wu J., Wang Y. (2024). Updated mechanisms of MASLD pathogenesis. Lipids Health Dis..

[45] Männistö V.T., Simonen M., Hyysalo J., Soininen P., Kangas A.J., Kaminska D., Matte A.K., Venesmaa S., Käkelä P., Kärjä V. (2015). Ketone body production is differentially altered in steatosis and non-alcoholic steatohepatitis in obese humans. Liver Int. Off. J. Int. Assoc. Study Liver..

[46] Lopaschuk G.D., Dyck J.R.B. (2023). Ketones and the cardiovascular system. Nat. Cardiovasc. Res..

[47] Yang H., Shan W., Zhu F., Wu J., Wang Q. (2019). Ketone Bodies in Neurological Diseases: Focus on Neuroprotection and Underlying Mechanisms. Front. Neurol..

[48] Neudorf H., Islam H., Falkenhain K., Oliveira B., Jackson G.S., Moreno-Cabañas A., Madden K., Singer J., Walsh J.J., Little J.P. (2024). Effect of the ketone beta-hydroxybutyrate on markers of inflammation and immune function in adults with type 2 diabetes. Clin. Exp. Immunol..

[49] Chen Y., Ouyang X., Hoque R., Garcia-Martinez I., Yousaf M.N., Tonack S., Offermanns S., Dubuquoy L., Louvet A., Mathurin P. (2018). β-Hydroxybutyrate protects from alcohol-induced liver injury via a Hcar2-cAMP dependent pathway. J. Hepatol..

[50] Luukkonen P.K., Qadri S., Ahlholm N., Porthan K., Männistö V., Sammalkorpi H., Penttilä A.K., Hakkarainen A., Lehtimäki T.E., Gaggini M. (2022). Distinct contributions of metabolic dysfunction and genetic risk factors in the pathogenesis of non-alcoholic fatty liver disease. J. Hepatol..

[51] Gambino R., Musso G., Cassader M. (2011). Redox balance in the pathogenesis of nonalcoholic fatty liver disease: Mechanisms and therapeutic opportunities. Antioxid. Redox Signal..

[52] Jung C.Y., Koh H.B., Heo G.Y., Ko B., Kim H.W., Park J.T., Yoo T.H., Kang S.W., Han S. (2024). . Association of ketone bodies with incident CKD and death: A UK Biobank study. Diabetes Metab..

[53] Flores-Guerrero J.L., Westenbrink B.D., Connelly M.A., Otvos J.D., Groothof D., Shalaurova I., Garcia E., Navis G., de Boer R.A., Bakker S.J.L. (2021). Association of beta-hydroxybutyrate with development of heart failure: Sex differences in a Dutch population cohort. Eur. J. Clin. Investig..

[54] Moore M.P., Shryack G., Alessi I., Wieschhaus N., Meers G.M., Johnson S.A., Wheeler A.A., Ibdah J.A., Parks E.J., Rector R.S. (2024). Relationship between serum β-hydroxybutyrate and hepatic fatty acid oxidation in individuals with obesity and NAFLD. Am. J. Physiol. Endocrinol. Metab..

[55] Bolinas D.K.M., Barcena A.J.R., Mishra A., Bernardino M.R., Lin V., Heralde F.C., Chintalapani G., Fowlkes N.W., Huang S.Y., Melancon M.P. (2025). Mesenchymal Stem Cells Loaded in Injectable Alginate Hydrogels Promote Liver Growth and Attenuate Liver Fibrosis in Cirrhotic Rats. Gels.

[56] Kasahara N., Teratani T., Doi J., Yokota S., Shimodaira K., Kaneko Y., Ohzawa H., Sakuma Y., Sasanuma H., Fujimoto Y. (2024). Controlled release of hydrogel-encapsulated mesenchymal stem cells-conditioned medium promotes functional liver regeneration after hepatectomy in metabolic dysfunction-associated steatotic liver disease. Stem Cell Res. Ther..

