# Supplementary material for: Plasma Beta-Hydroxybutyrate and All-Cause Mortality in Patients with Liver Cirrhosis
Source: Biomedicines. 2025 May 6;13(5):1120. doi: 10.3390/biomedicines13051120 (PMC12109306; doi:10.3390/biomedicines13051120)
Supplement: Supplementary file 1 [file biomedicines-13-01120-s001.zip › Supplementary Figures and Tables BHB Cirrhosis.pdf]

Supplementary Material:

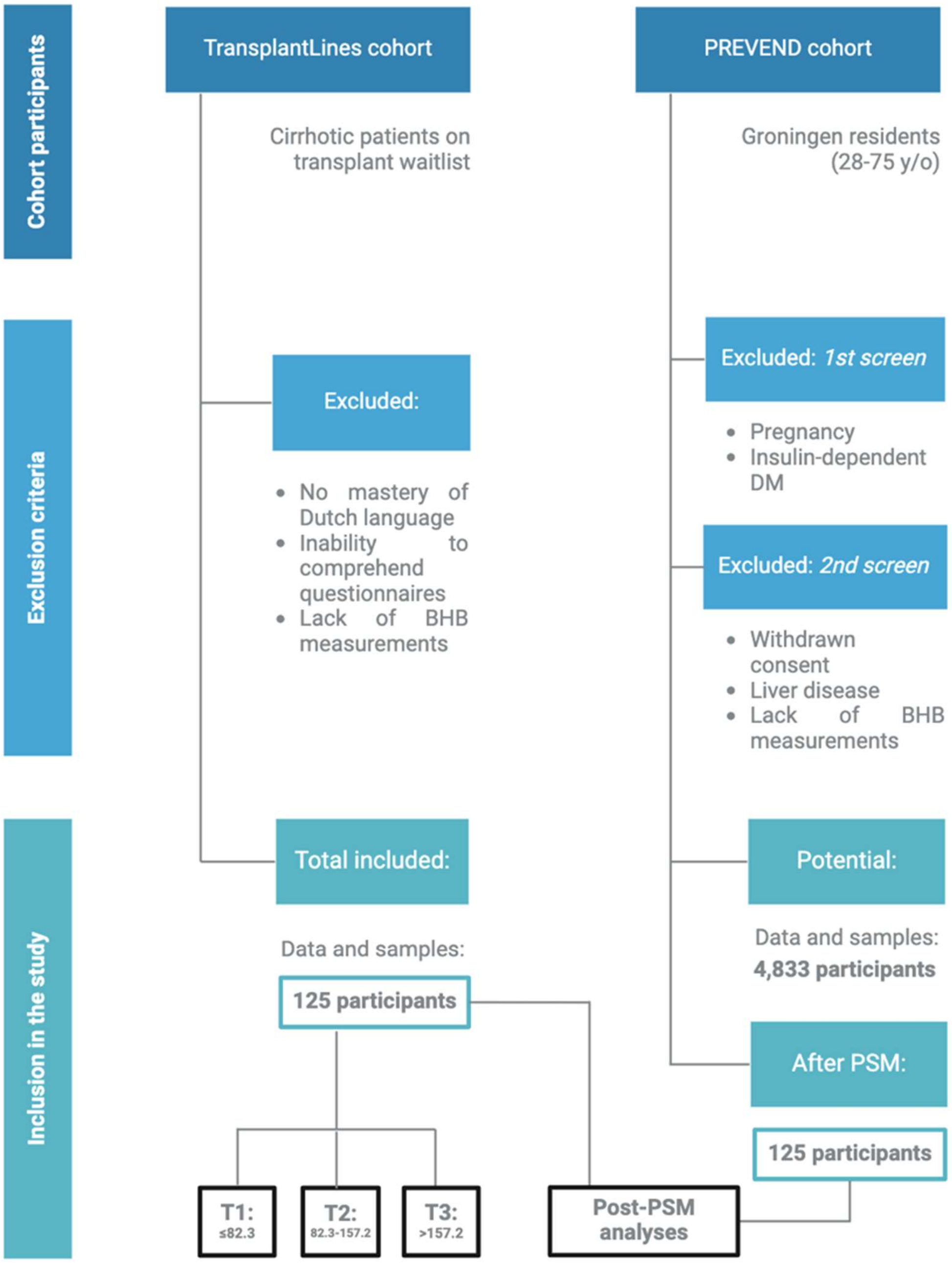

Supplementary Figure 1. Participant selection flow-chart. BHB: β-hydroxybutyrate, DM: Diabetes mellitus, PSM: Propensity-score matching; T1-T3: Tertiles 1-3 (values shown in µmol/L).

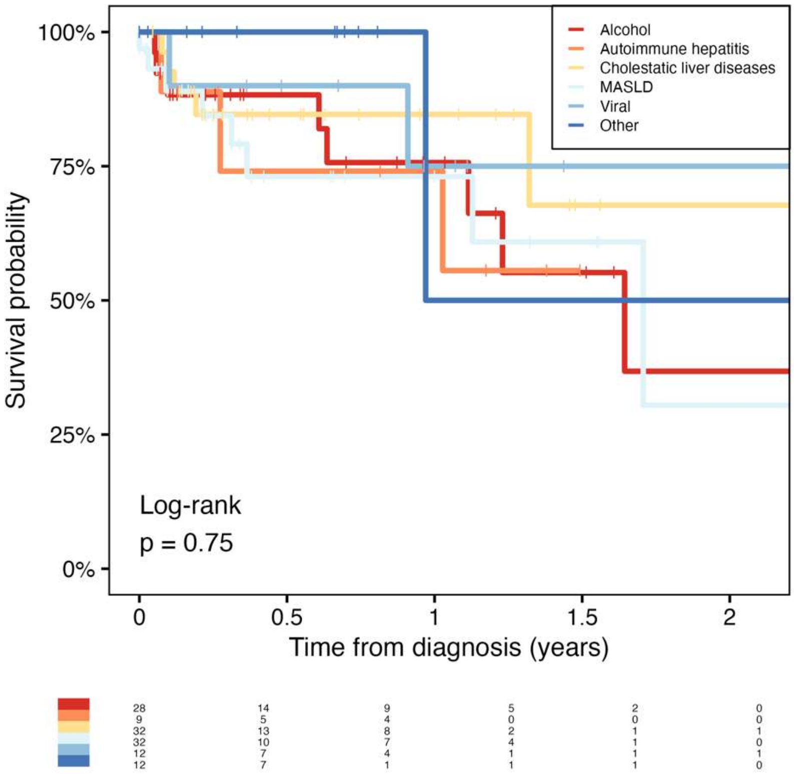

Supplementary Figure 2. Kaplan-Meier survival curves for the association between different etiologies of cirrhosis and the risk of all-cause mortality in end-stage liver disease patients on waiting list for LT. “Other” etiologies refer to storage diseases, vascular etiologies and biliary atresia, which were grouped given their small group sizes.

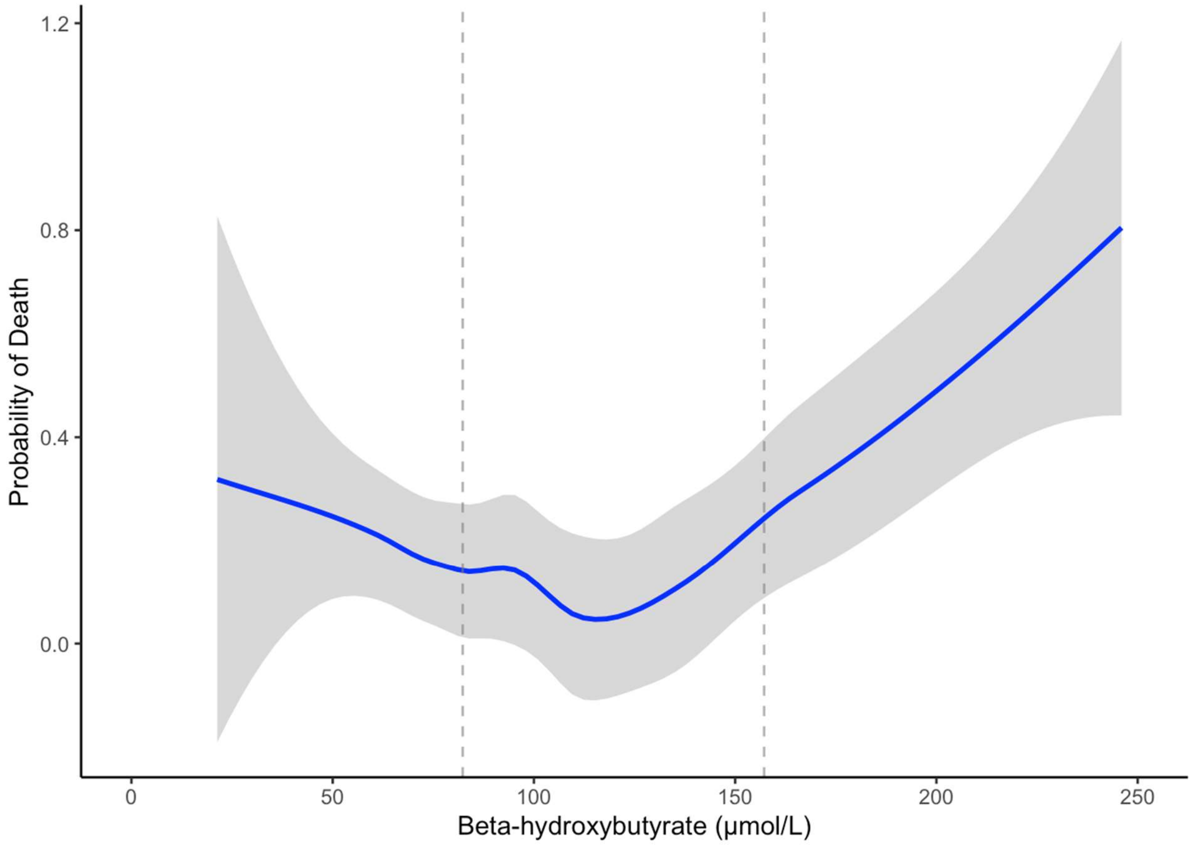

Supplementary Figure 3: LOESS curve (with 95 % confidence intervals) showing the relationship of BHB and the probability of death in patients with cirrhosis. The vertical dashed lines at 82.3 µmol/L and 157.2 µmol/L represent the tertile cutoffs for BHB.

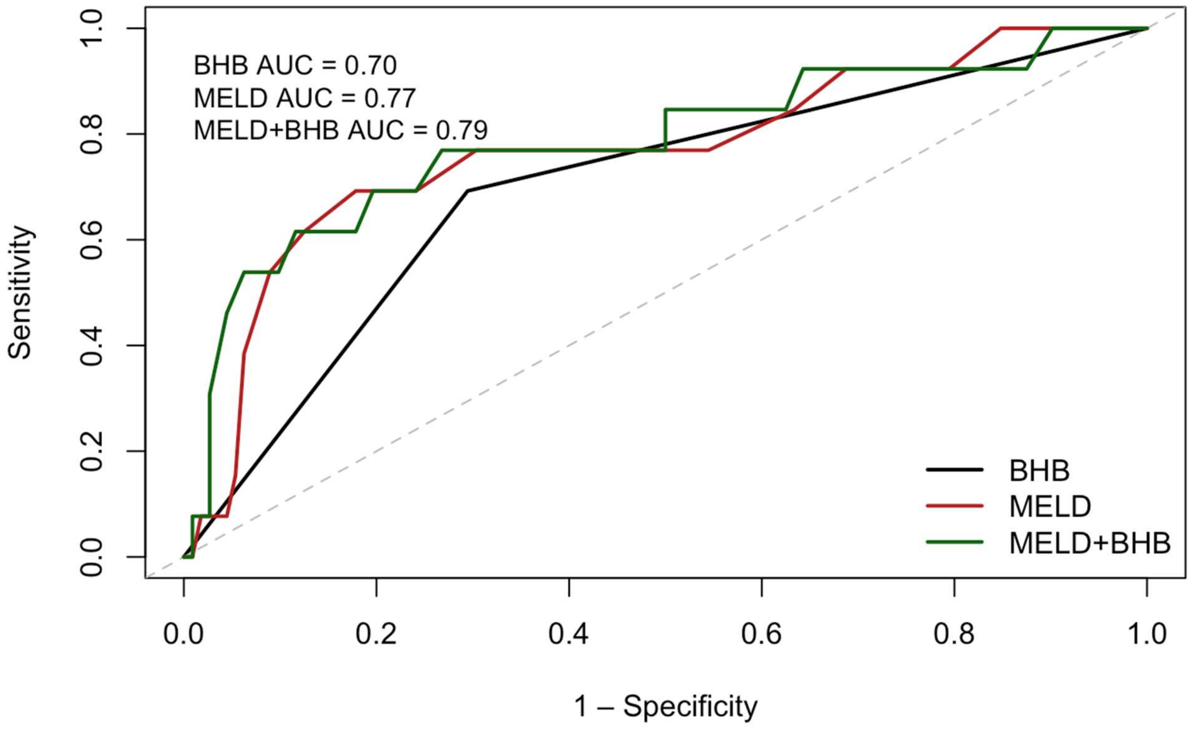

Supplementary Figure 4: Receiver operating characteristic (ROC) curves for predicting 90-day all-cause mortality in patients with cirrhosis, comparing discrimination performance between plasma beta-hydroxybutyrate (BHB), MELD score, and the combined model of MELD+BHB. Area under the ROC curve (AUC) values for BHB is 0.70 (95% CI: 0.55-0.85), for MELD is 0.77 (95% CI: 0.61-0.92), and for MELD+BHB is 0.79 (0.63-0.95).

Supplementary Tables

Table S1: Clinical and laboratory characteristics in cirrhotic and all PREVEND participants

	**Cirrhosis (n = 125)**	**PREVEND (n = 4,833)**	***p***
**β -hydroxybutyrate (μmol/L)**	111.5 [75.9, 178.1]	121.8 [93.1, 169.1]	0.1
**Age (years)**	60 [52, 65]	53 [44., 63]	<0.001
**Sex (Female; N, %)**	42 (33.6)	2447 (50.6)	<0.001
**BMI (kg/m^2)**	27.8 [24.8, 30.9]	26.1 [23.8, 28.9]	<0.001
**Smoking (N, %)**	16 (12.8)	1320 (27.3)	<0.001
**Alcohol consumption (gr/day; N, %)**			<0.001
**0**	120 (96.0)	1695 (35.1)	
**0.1-10**	5 (4.0)	1217 (25.2)	
**10-30**	0 (0.0)	993 (20.5)	
**>30**	0 (0.0)	928 (19.2)	
**Systolic Blood Pressure (mmHg)**	115 [107, 130]	123 [112, 137]	0.001
**Diastolic Blood Pressure (mmHg)**	65 [59, 75]	72 [67, 79]	<0.001
**History of cardiovascular disease (N, %)**	6 (4.8)	301 (6.2)	0.6
**History of diabetes (N, %)**	35 (28.0)	294 (6.1)	<0.001
**Antihypertensive drugs (N, %)**	79 (63.2)	854 (17.7)	<0.001
**Glucose-lowering drugs (N, %)**	34 (27.2)	178 (3.7)	<0.001
**Lipid-lowering drugs (N, %)**	19 (15.2)	457 (9.5)	0.046
**Fasting glucose (mmol/L)**	6.35 [5.03, 8.00]	4.80 [4.40, 5.30]	<0.001

**eGFR (ml/min/1.73m^2)**	99.50 [76.10, 109.70]	93.70 [81.62, 104.29]	0.1
**Total cholesterol (mmol/L)**	3.26 [2.61, 4.14]	5.34 [4.71, 6.08]	<0.001
**HDL cholesterol (mmol/L)**	0.88 [0.59, 1.19]	1.21 [1.03, 1.43]	<0.001
**LDL cholesterol (mmol/L)**	1.81 [1.29, 2.25]	3.50 [2.93, 4.14]	<0.001
**Triglycerides (mmol/L)**	0.67 [0.46, 1.07]	1.11 [0.81, 1.60]	<0.001
**ALT (U/L)**	39 [28, 59]	17 [13, 24]	<0.001
**AST (U/L)**	54 [44, 83]	22 [19, 26]	<0.001
**GGT (U/L)**	95 [49, 151]	24 [16, 38]	<0.001
**AP (U/L)**	141 [99, 210]	66 [55, 79]	<0.001
**Total Bilirubin (mmol/L)**	40 [23, 94]	7 [5, 9]	<0.001
**Hemoglobin (mmol/L)**	6.9 [5.9, 7.8]	8.5 [8.0, 9.0]	<0.001

Data are expressed in median (IQR) for continuous variables and in numbers (N) and percentages for categorical variables. *p*-values by Mann-Whitney U test for numeric variables and Chi-squared test for categorical variables. ALT: alanine aminotransferase, AP: alkaline phosphatase, AST: aspartate aminotransferase, BMI: body-mass index, eGFR: estimated glomerular filtration rate, GGT: gamma-glutamyl transferase, HDL: high-density lipoprotein, LDL: low-density lipoprotein.

Table S2: Univariable and multivariable linear regression analysis showing association between BHB and relevant clinical and laboratory parameters in patients with cirrhosis

	**Univariable**		**Multivariable**	
	**Std. β**	***p*-value**	**Std. β**	***p*-value**
**Age**	0.068 (-0.108, 0.244)	0.452		
**BMI**	-0.092 (-0.268, 0.084)	0.310		
**Diabetes**	**0.43 (0.045, 0.815)**	**0.03**	**0.456 (0.078, 0.833)**	**0.020**
**Antihypertensive drugs**	0.19 (-0.173, 0.553)	0.308		
**Glucose-lowering drugs**	0.237 (-0.156, 0.631)	0.239		
**Lipid-lowering drugs**	-0.228 (-0.716, 0.261)	0.363		
**eGFR**	-0.019 (-0.196, 0.157)	0.831		
**SBP**	0.01 (-0.217, 0.236)	0.934		
**DBP**	0.05 (-0.176, 0.277)	0.666		
**CTP classification**	-0.056 (-0.232, 0.121)	0.537		
**MELD score**	0.075 (-0.101, 0.252)	0.403		
**HbA1c**	0.568 (0.237, 0.899)	0.001		
**Fasting glucose**	0.202 (-0.004, 0.408)	0.063		

**Total cholesterol**	0.036 (-0.14, 0.213)	0.689		
**HDL cholesterol**	**-0.202 (-0.375, -0.029)**	**0.024**	**-0.213 (-0.383,-0.043)**	**0.016**
**LDL cholesterol**	-0.022 (-0.199, 0.154)	0.806		
**Triglycerides**	0.168 (-0.006, 0.342)	0.061		
**ALT**	0.052 (-0.192, 0.296)	0.678		
**AST**	0.072 (-0.172, 0.316)	0.564		
**GGT**	0.125 (-0.118, 0.368)	0.316		
**AP**	-0.017 (-0.261, 0.228)	0.894		
**Total Bilirubin**	0.185 (-0.048, 0.418)	0.124		
**Albumin**	0.055 (-0.188, 0.299)	0.658		

One multivariable model is shown adjusting for Diabetes and HDL cholesterol. ALT: alanine aminotransferase, AP: alkaline phosphatase, AST: aspartate aminotransferase, BMI: body-mass index, CTP: Child-Turcotte-Pugh, DBP: diastolic blood pressure, eGFR: estimated glomerular filtration rate, GGT: gamma-glutamyl transferase, HbA1c: glycated hemoglobin, HDL: high-density lipoprotein, LDL: low-density lipoprotein, MELD: model for end-stage liver disease, SBP: systolic blood pressure, Std. β: standardized regression coefficients.

Table S3: Cox regression analyses for associations between plasma BHB levels and the risk of all-cause mortality in patients with cirrhosis, excluding rare etiologies

	**T1<br>HR [95% CI]**	**T2<br>(Reference)**	**T3<br>HR [95% CI]**
**Sensitivity analysis: Excluding patients with rare etiologies (n = 104, deaths = 23)**			
**Model 1**	1.2 [0.4-4.3]<br>p = 0.7	**Reference**	**4.3 [1.4-13.4]<br>p = 0.01**
**Model 2**	1.2 [0.4-4.3]<br>p = 0.7	**Reference**	**4.5 [1.4-14.4]<br>p = 0.01**
**Model 3**	1.4 [0.4-5.0]<br>p = 0.6	**Reference**	**3.6 [1.1-11.6]<br>p = 0.03**
**Model 4**	1.0 [0.3-3.8]<br>p = 0.9	**Reference**	**3.6 [1.1-11.9]<br>p = 0.04**
**Model 5**	1.2 [0.3 -4.8]<br>p = 0.8	**Reference**	**3.4 [1.1-11.2]<br>p = 0.04**

Model 1: crude model. Model 2: adjusted for age and sex. Model 3, adjusted for age, sex and MELD score. Model 4: adjusted for age, sex, MELD score and history of diabetes. Model 5: adjusted for age, sex, MELD score, history of diabetes and HDL cholesterol. T1-T3: tertiles 1-3; HR: hazard ratio; CI: confidence interval; MASLD: metabolic-dysfunction associated steatotic liver disease; Rare etiologies: vascular etiologies, autoimmune hepatitis, storage diseases and biliary atresia

Table S4: Cox regression analyses for associations between plasma BHB levels and the risk of all-cause mortality in cirrhotic patients, between T1 and T3			
	T1	T3	
		HR［95%CI］	*p*-value
**All cirrhotic patients**			
**Model 1**	Reference	**3.64［1.51-8.77］**	***p* = 0.004**
**Model 2**	Reference	**3.53［1.45-8.58］**	***p* = 0.006**
**Model 3**	Reference	**2.57［1.01-6.55］**	***p* = 0.048**
**Model 4**	Reference	**3.11［1.10-8.80］**	*p* **= 0.032**
**Model 5**	Reference	2.75［0.96-7.86］	*p* = 0.059
**Sensitivity analysis: Excluding diabetic patients**			
**Model 1**	Reference	**5.72［1.95-16.80］**	***p* = 0.001**
**Model 2**	Reference	**5.32［1.80-15.76］**	***p* = 0.003**
**Model 3**	Reference	**3.58［1.08-11.83］**	***p* = 0.037**
**Model 4’**	Reference	2.64［0.77-9.11］	*p* = 0.125
**Sensitivity analysis: Excluding MASLD patients**			
**Model 1**	Reference	**3.59［1.26-10.20］**	***p* = 0.017**
**Model 2**	Reference	**3.26［1.14-9.35］**	***p* = 0.028**
**Model 3**	Reference	2.33［0.77-7.09］	*p* = 0.137
**Model 4**	Reference	**3.31［1.01-10.87］**	***p* = 0.049**
**Model 5**	Reference	2.52［0.69-9.23］	*p* = 0.164

Model 1: crude model.

Model 2: adjusting for age and sex.

Model 3, adjusting for age, sex and MELD score.

Model 4: adjusting for age, sex, MELD score and history of diabetes.

Model 5: adjusting for age, sex, MELD score, history of diabetes and HDL cholesterol.

Model 4’: in sensitivity analysis excluding diabetic patients model 4 was adjustment for age, sex, MELD score and HDL cholesterol

T1-T3: tertiles 1-3, HR: hazard ratio, CI: confidence interval, MASLD: metabolic-dysfunction associated steatotic liver disease.
